# Effect of methyl DNA adducts on 3’-5’ exonuclease activity of human TREX1

**DOI:** 10.1042/BCJ20240600

**Published:** 2025-03-05

**Authors:** Nikhil Tuti, Unnikrishnan P. Shaji, Susmita Das, Roy Anindya

**Affiliations:** 1 Department of Biotechnology, Indian Institute of Technology Hyderabad (IITH), Telangana, Kandi, Sanga Reddy 502284, India

**Keywords:** DNA damage, exonuclease, 1-methyladenine, 3-methylcytosine, TREX1

## Abstract

Three-prime repair exonuclease 1 (TREX1) is a 3′-5′ exonuclease that plays an important role in clearing cytoplasmic DNA. Additionally, TREX1 is translocated to the nucleus after DNA damage and assists in DNA repair. In this work, we evaluated the activity of TREX1 in the context of the removal of methyl DNA adducts. We observed that TREX1 was less efficient at degrading methyl methanesulfonate (MMS)-treated methylated DNA compared with normal DNA. Two methyl DNA adducts, N1-methyladenine and N3-methylcytosine, were found to block TREX1 exonuclease activity. To understand the mechanism of limited TREX1-mediated degradation of MMS-damaged DNA, stem-loop substrates containing solitary methyl adducts were prepared. We found that when the solitary methyl adducts were present at the 3′-terminal single-stranded overhang, it prevented degradation by TREX1. However, TREX1 could efficiently process internally located duplex DNA methyl adducts when the 3′-terminal of the scissile strand was damage-free. Broadly, these observations suggest that TREX1 may be capable of resecting methyl adducts containing DNA, but it might be less proficient of removing 3′-terminal DNA methyl adducts.

## Introduction

Exposure to endogenous alkylating agents [1] and aberrant DNA methyltransferase (DNMT) activity can lead to multiple alkyl DNA adducts [2]. Some of the DNA methyl adducts, including N-7-methylguanine (7 mG), N-7-methyladenine, N-3-methylguanine, and N-3-methyladenine, destabilize the nucleobase structure leading to spontaneous removal [3]. Some methyl DNA adducts, such as *N-*1-methyladenine (1mA) and *N-*3-methylcytosine (3mC), are considered cytotoxic as they disrupt Watson–Crick hydrogen bonding between DNA base pairs and hinder DNA replication [4,5]. Methylating agents can react with DNA at 12 different sites on the DNA bases, especially all the exocyclic oxygens and ring nitrogens [6]. One of the agents, methyl methanesulfonate (MMS) predominantly produces 7 mG, 1mA, and 3mC. Among these adducts, 3mC and 1mA are predominantly repaired by the two members of Fe(II)/2-oxoglutarate(2OG)-dependent dioxygenase family, known as ALKBH2 (UniProtKB Q6NS38) and ALKBH3 (UniProtKB Q5XIC8) [7]. If left unrepaired, 3mC completely blocks Pol β and Pol λ and partially blocks Pol δ [8]. It remains unknown whether these lesions at the DNA 3′-terminal are inhibitory to any 3′-5′ exonucleases. One of the exonucleases that might generate a ‘clean’ 3′ hydroxyl and play a role in DNA repair is three-repair exonuclease 1 (TREX1, UniProtKB Q9NSU2). TREX1 was also shown to process DNA 3′-termini with mismatched 3′-overhang [9–12], 3′-phospho-α,β-unsaturated aldehyde [13], O4-methylthymine, O6-methylguanine, a deaminated base such as uracil and hypoxanthine [14,15] but not abasic sites, 3′-phosphate, 3′-phosphoglycolate [16], and UV photoproducts [17]. Although the base preferences for the TREX1 and related DEDDh exonuclease family were also studied previously [13–15,18–20], the effect of N-methylated base on TREX1 was not studied. The role of TREX1 in the nucleus remains poorly understood, although it was shown to co-operate with polβ *in vitro* in a reconstituted human base excision repair (BER) reaction [21]. The N-terminal region of TREX1 contains nuclease domain, and the C-terminal region has a transmembrane domain (TMD). This TMD anchors TREX1 at the ER and normally prevents TREX1 from interacting with genomic DNA, unless there is a genomic stress [22]. In the cytoplasm, TREX1 degrades cytosolic DNA, thus suppressing aberrant cGAS-STING-type-I interferon signalling [23,24]. Loss-of-function mutations in human *TREX1* are associated with a broad range of autoimmune and inflammatory diseases, and the disease phenotype is related to cGAS-STING activation and cytokine induction as a result of cytosolic DNA accumulation [25]. *Trex1^−/−^* mice developed inflammatory myocarditis, and the disease phenotype of TREX1 deficiency was so severe that it is probably difficult to fully exclude a concomitant role for TREX1 in regulating DNA damage repair [26]. Interestingly, recent studies showed that the deletion of *TREX1* accelerates spontaneous cellular senescence and activates the DNA damage response [27]. Based on these reports, we thought it would be worthwhile to examine whether TREX1 could remove cytotoxic methyl adducts, especially 1mA and 3mC. In this report, we show that these adducts inhibit TREX1 activity, especially when present at the 3′-overhang section of the scissile strand.

## Results

### MMS-treated methylated DNA significantly inhibits TREX1

**Figure 1:**
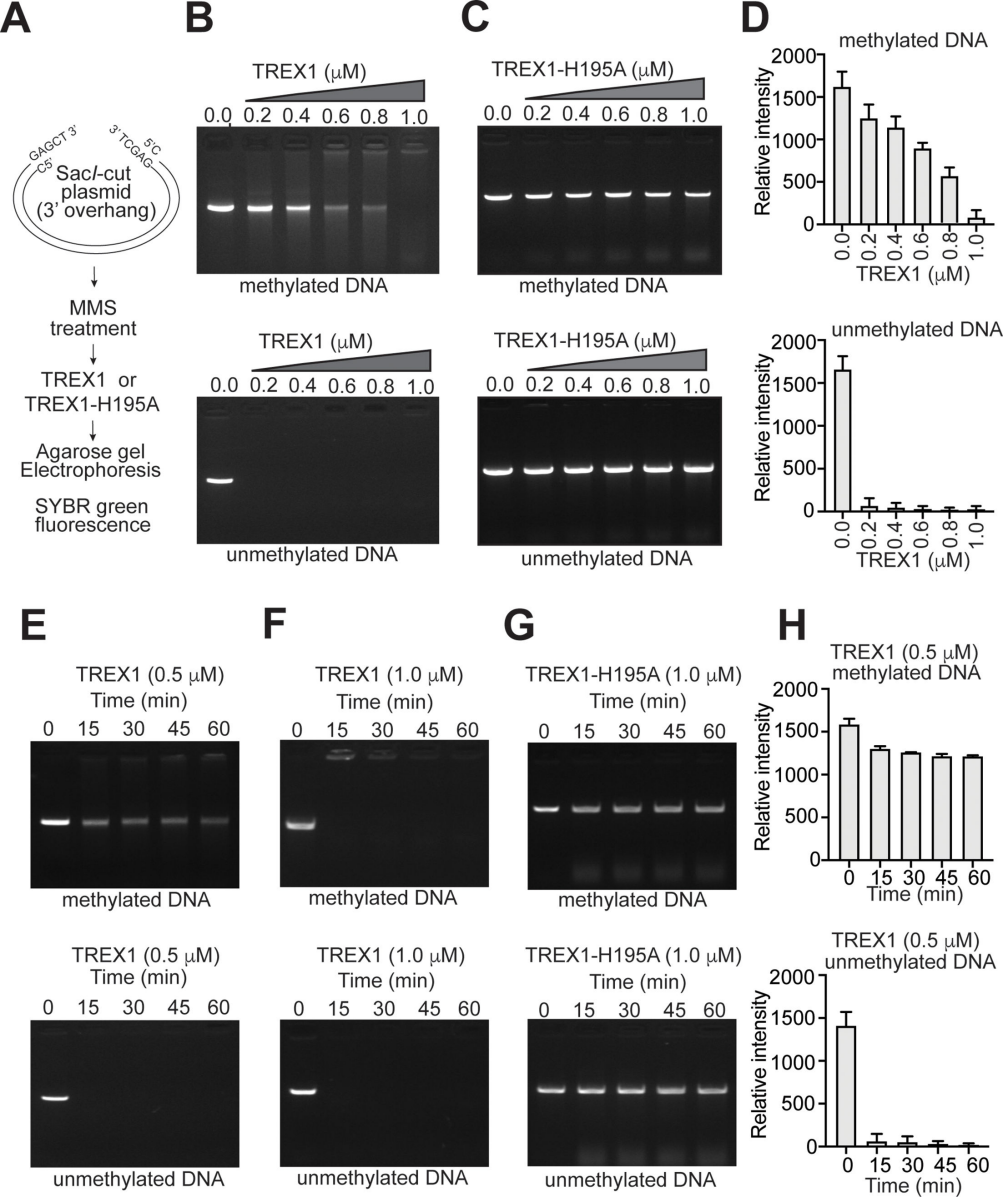
Degradation of DNA containing methyl adducts by TREX1. (**A**) Schematic outline of preparation of methylated and unmethylated substrate DNA for TREX1. (**B**) Agarose gel electrophoretic analysis of the exonuclease activities of the recombinant wild type TREX1. The assay was performed using Sac*I*-cut pBluscript DNA (unmethylated) or MMS-treated (methylated) DNA and increasing concentration of wild-type TREX1. (**C**) The exonuclease activities of the recombinant catalytically inactive TREX1 H195A mutant were tested as depicted in B. (**D**) Comparison of DNA degradation by wildtype TREX1 using methylated and unmethylated linearized plasmid DNA as depicted in B. (**E**) Time-course of TREX1 activity using unmethylated (bottom) or methylated DNA (top) substrate and wild type TREX1 (0.5 μg). (**F**) Time-course of TREX1 activity using a higher concentration of wild type TREX1 (1.0 μg). (**G**) Time -course of DNA degradation by wildtype and mutant TREX1 using methylated and unmethylated linearized plasmid DNA. (**H**) Time-course of DNA degradation by wildtype TREX1 using methylated and unmethylated linearized plasmid DNA as depicted in E. All data represent mean ± S.D. (error bars) from triplicate experiments. TERX1, three-prime repair exonuclease 1.

A commonly used cytotoxic laboratory alkylating agent MMS produces high proportions of 7meG and cytotoxic methyl adducts such as 1mA and 3mC [28]. Since TREX1 was shown to degrade and clear DNA from dying cells [29], it would be of interest to characterize whether TREX1 can digest DNA damaged by MMS *in vitro*. As TREX1 was reported to preferably degrade 3′-overhang-containing DNA compared with blunt-end DNA or DNA with 5′-overhang [12], we linearized plasmid DNA by digesting with *SacI*. This linearized duplex DNA containing 3′-overhang was treated with MMS to generate various methyl adducts (Figure 1A). Purified truncated recombinant TREX1 and catalytic mutant TREX1-H195A (Supplementary information) were incubated with these DNA substrates, and DNA degradation was measured by gel electrophoresis. In the presence of increasing concentration of wildtype TREX1, unmethylated DNA was undetectable (Figure 1B, bottom panel), whereas the methyl adduct-containing reaction product was partially detectable (Figure 1B, top panel). We also noticed high molecular mass DNA bands on methylated plasmid DNA. This might be due to denaturation of DNA during MMS treatment. As expected, catalytically inactive mutant TREX1-H195A showed no activity and failed to degrade either unmethylated or methylated DNA (Figure 1C, top and bottom panel). We repeated this experiment multiple times (Figure 1D) and found the results reproducible. These data clearly suggest that TREX1 has limited activity on methylated DNA, and degradation can only be seen when TREX1 was used at higher concentrations. The effect of incubation time on TREX1 activity was also investigated. A longer time was required to degrade MMS-treated methylated DNA (Figure 1E, top panel). Only when the exonuclease concentration was increased, MMS-treated DNA was degraded (Figure 1F, top panel). Notably, unmethylated DNA was rapidly degraded by TREX1 (Figure 1E and F, bottom panel,and Figure 1H). To confirm that DNA is degraded due to exonuclease activity, reactions were performed including TREX1 H195A mutant (Figure 1G, top and bottom panel). Catalytically inactive mutant TREX1 failed to degrade both methylated and unmethylated DNA. These data suggest that MMS-induced DNA methyl adducts substantially inhibit TREX1 activity, and only a high concentration of TREX1 or much longer incubation with TREX1 could digest MMS-damaged DNA.

**Figure 2:**
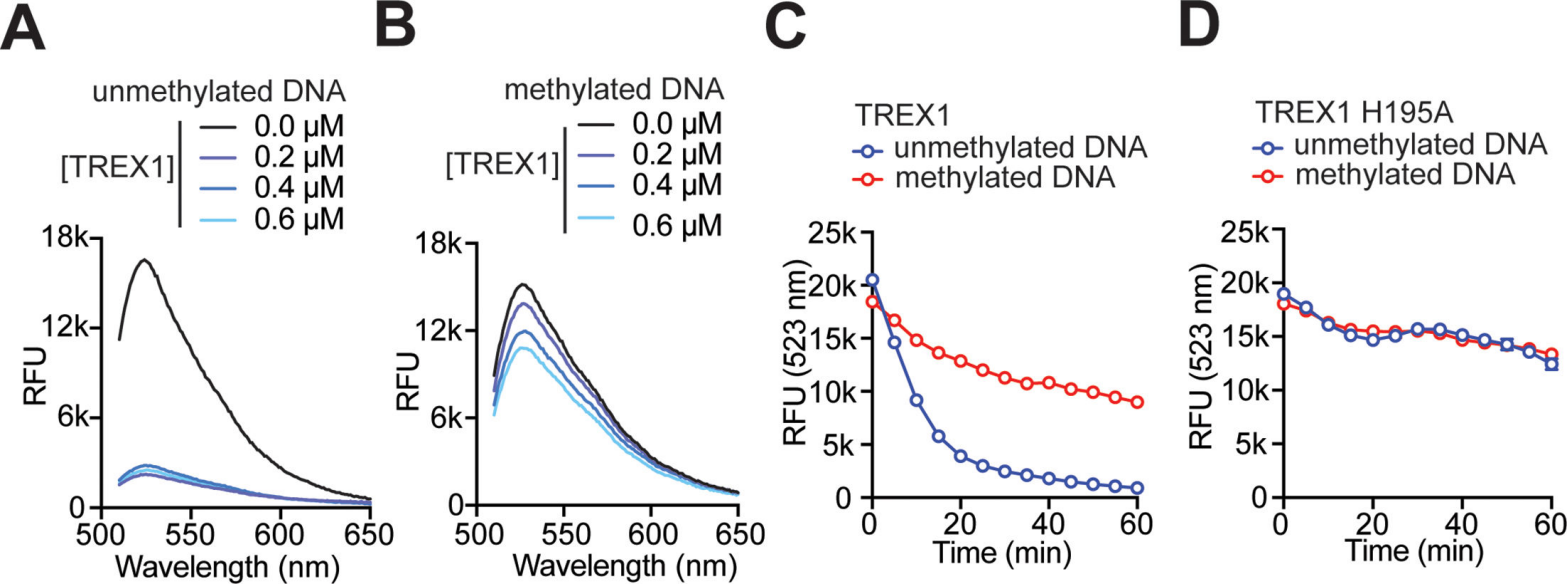
Effect of methyl adducts on the exonuclease activities of the TREX1 analysed by SYBR Green (SG)-based DNA fluorescence. An increasing concentration of wildtype TREX1 was used against Sac*I*-cut pBluscript DNA (unmethylated DNA) and MMS-treated DNA (methylated DNA). (**A**) uUnmethylated DNA was incubated with TREX1, and SG fluorescence spectra were analysed. (**B**) MMS-treated methylated DNA was incubated with TREX1, and DG fluorescence spectra were analysed. Time-dependent degradation was analysed by SG-based DNA fluorescence using TREX1 (**C**) and mutant H195A TREX1 (**D**). Comparative analysis of DNA degradation expressed by reduction ofin SG fluorescence. All data represent mean ± S.E. (error bars) from five independent experiments. TERX1, three-prime repair exonuclease 1.

To further validate the result, a fluorescence-based assay was performed that offers a real-time analysis of TREX1 activity [30]. For this, *SacI*-cut plasmid DNA having a 3′-overhang was fluorescently labelled with SYBR green (SG) [31] and incubated with TREX1 and TREX1 H195A mutant. Because the SG fluorescence signal is directly related to the amount of duplex DNA, any decrease in SG fluorescence would indicate TREX1-catalysed DNA degradation. We observed that the fluorescence signal reduced significantly in the presence of undamaged DNA (Figure 2A). The reduction in fluorescence required a significantly higher concentration of TREX1 to degrade MMS-treated methylated DNA (Figure 2B). The time-dependent gradual decrease in the fluorescence signal for methylated and unmethylated DNA showed that the reduction in fluorescence was significantly slower in the presence of methylated DNA (Figure 2C). The control experiment with catalytic mutant TREX1 showed a failure to degrade both methylated and unmethylated DNA (Figure 2D). These results confirmed our results from gel electrophoresis analysis (Figure 1) that TREX1 needs a higher concentration or longer reaction time to degrade MMS-treated DNA compared with unmethylated DNA. Thus, methylated DNA appeared to be a poorer substrate for TREX1. The fact that the presence of methyl DNA adducts in the DNA could substantially abrogate its ability to serve as a substrate for TREX1 prompted us to investigate the nature of the adducts that were inhibitory to TREX1 and the mechanism of low level of activity of TREX1 activity against MMS-treated methylated DNA.

### Identification of methyl adducts affecting the exonuclease activity of TREX1

**Figure 3:**
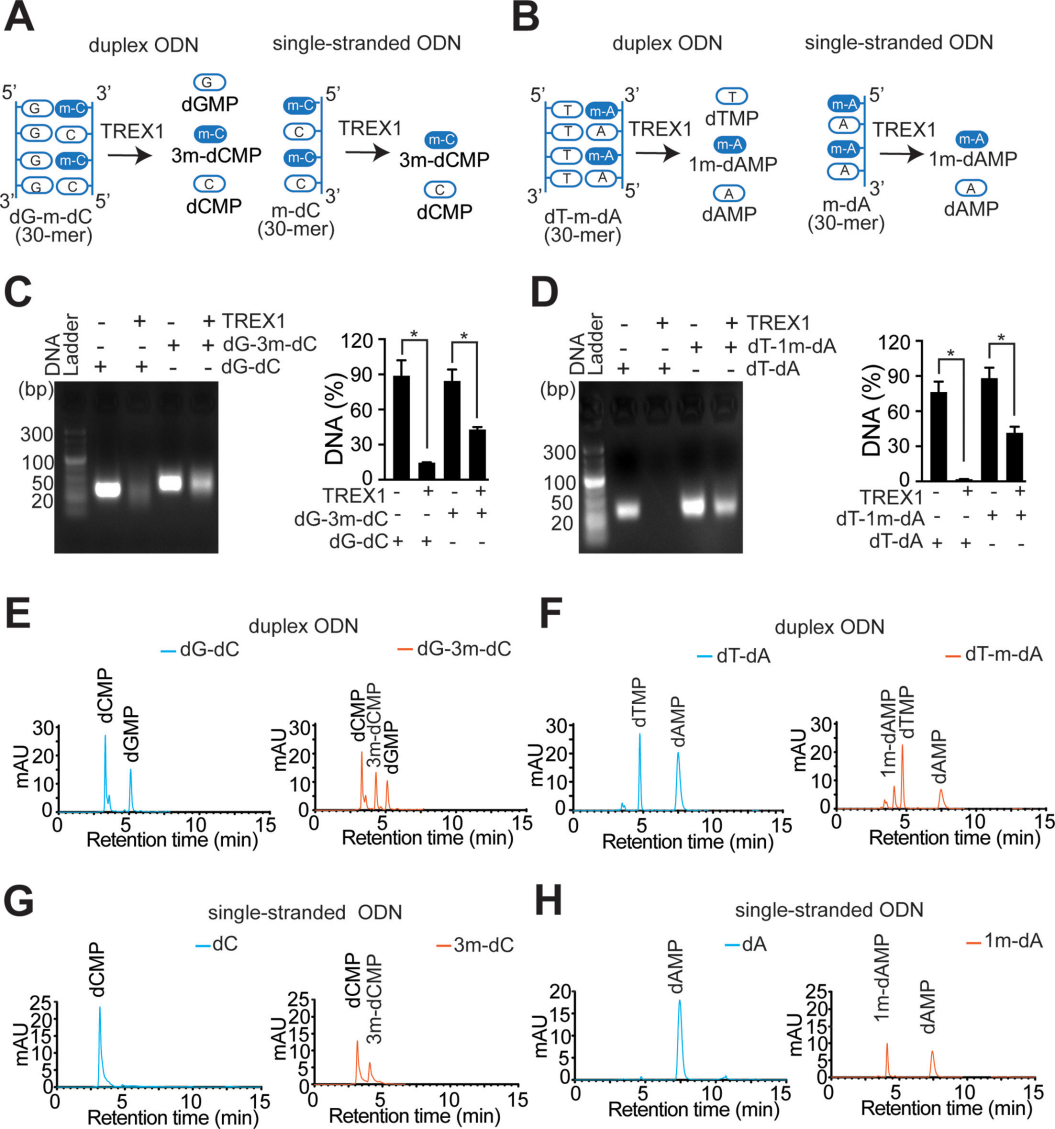
Effect of 3mC and 1mA adducts on the exonuclease activity of TREX1. The schematic design of linear DNA duplex oligonucleotide (ODN) or single-stranded ODN substrate containing alternate 3m-dC and normal dC (**A**) or alternate 1 m-dA and 1 m-dA (**B**). The nucleoside monophosphate expected to form TREX1-mediated degradation is also depicted. The resulting nucleotides were analysed by agarose gel electrophoresis or liquid chromatography (HPLC). (**C**) Agarose gel electrophoretic analysis of TREX1-mediated degradation of duplex ODN containing multiple 3mC (left panel) and its comparative analysis (right panel). (**D**) Agarose gel electrophoretic analysis of TREX1-mediated degradation of duplex ODN containing multiple 1mA (left panel) and its comparative analysis (right panel). (**E**) HPLC analysis of TREX1-mediated degradation of duplex ODN containing normal dC-dG (left panel) or alternate 3m-dC and normal dC (right panel). Duplex ODN substrates were prepared by annealing 30-mer dG strand to the complementary strand containing dC or 3m-dC. (**F**) HPLC analysis of TREX1-mediated degradation of duplex ODN containing normal dA-dT (left panel) or alternate 1 m-dA and normal dA (right panel). Duplex ODN substrates were prepared by annealing 30-mer dT strand to the complementary strand containing dA or 1 m-dA. (**G**) HPLC analysis of TREX1-mediated degradation of single-stranded 30-mer ODN containing normal dC (left panel) or 3m-dC (right panel). (**H**) HPLC analysis of TREX1-mediated degradation of single-stranded 30-mer ODN containing normal dA (left panel) or 1 m-dA (right panel). 1mA, *N-*1-methyladenine; 3mC, *N-*3-methylcytosine; TERX1, three-prime repair exonuclease 1.

Treatment with MMS results in 12 different types of methyl DNA adducts, including 7meG, 3meA, 1mA, and 3mC. Adopting a candidate approach, we investigated two distinct adducts that were reported to be cytotoxic, namely, 1mA and 3mC. For the ssDNA substrate, synthetic oligonucleotides containing alternate 1mA and A or 3mC and C were taken. For duplex DNA, synthetic DNA containing alternate 1mA and A (or 3mC and C) was annealed with oligo-dT (or oligo-dG) (Figure 3A and B). The TREX1 activity was evaluated by gel electrophoresis analysis. Compared with the undamaged DNA oligonucleotides, more DNA containing 3mC or 1mA adducts was detectable (Figure 3C and D). These results showed that DNA with multiple 3mC or 1mA adducts is poorly processed by TREX1.

The evaluation of the ability of TREX1 in removing 3mC or 1mA as methylated nucleotide monophosphates (d-3m-CMP or d-1m-AMP) from single-stranded and duplex DNA was monitored by analysing the degradation products using high-performance liquid chromatography(HPLC). It was observed that the level of nucleotides monophosphates (dCMP, 3m-dCMP, and dGMP) from methylated duplex oligonucleotide was lower than that of unmodified duplex oligonucleotide control (dAMP and dTMP) (Figure 3E). Similarly, TREX1 was inefficient in digesting duplex oligonucleotides containing multiple 1mA as observed from the level of nucleotide monophosphates (dAMP, 1m-dAMP, and dTMP) (Figure 3F). As reported previously, exonuclease TREX1 also degrades ssDNA [32]. We tested its ability to remove multiple 3mC or 1mA adducts from the ssDNA. Results revealed that ssDNA containing 1mA and 3mC was poorly degraded by TREX1, compared with unmethylated oligo-dA (Figure 3G and H). Together, it can be concluded that the limited efficiency of TREX1 against MMS-treated methylated DNA might be explained by the poor activity of TREX1 against 3mC or 1mA.

### Effect of position of DNA methyl adducts on TREX1 activity

**Figure 4:**
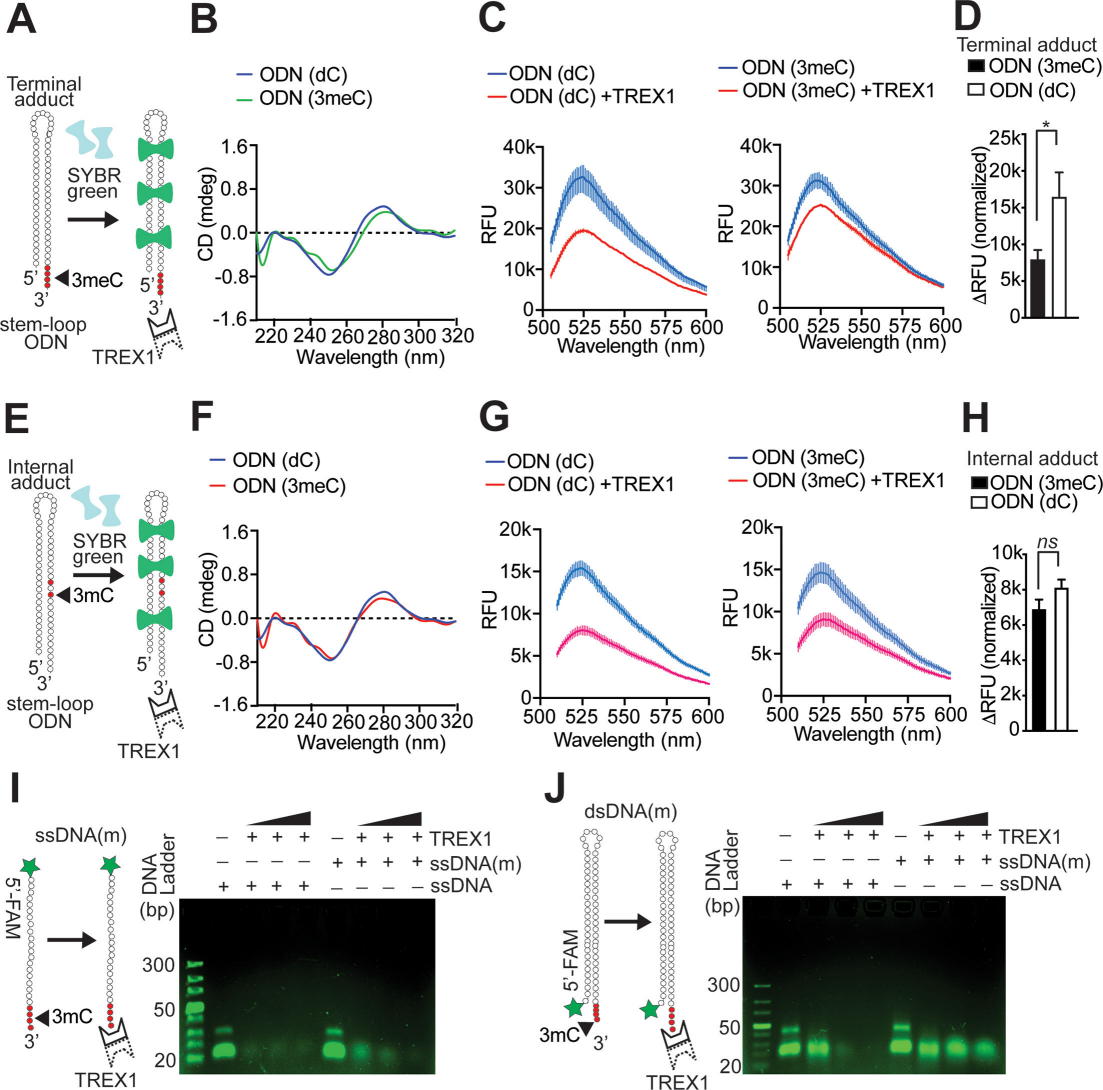
Effect of position of 3mC adduct on the exonuclease activity of TREX1. (**A**) The design of duplex DNA with a stem-loop oligonucleotide (stem-loop ODN) substrate containing 3′-terminal 3m-dC. The duplex structure had 37 base pairs connected by a 9-bp linker. A schematic outline of the SG fluorescence-based experiment is also depicted. (**B**) Comparative CD spectra of stem-loop ODN substrate with or without terminal 3mC. (**C**) Effect of 3 ′-terminal 3mC on TREX1 activity. SG-bound stem-loop ODN having terminal 3m-dC or normal dC wereas incubated with TREX1, and fluorescence spectra were analysed. (**D**) Comparative analysis of DNA degradation expressed by reduction ofin SG fluorescence. (**E**) The design of stem-loop ODN substrate containing internal 3m-dC. A schematic outline of the SG fluorescence-based experiment is also depicted. (**F**) Comparative CD spectra of stem-loop ODN substrate containing internal 3m-dC. (**G**) Effect of internal 3m-dC on TREX1 activity. SG-bound stem-loop ODN having internal 3m-dC or normal dC wereas incubated with TREX1, and fluorescence spectra were analysed (**H**) Comparative analysis of DNA degradation expressed by reduction ofin SG fluorescence. (**I**) The schematic design of single-stranded DNA containing 3mC at the 3′ -terminus and fluorescently- labelled (FAM) at the 5 ′-terminus is depicted as ssDNA(m). Single-stranded DNA without 3mC at the 3′ -terminus and FAM-labelled at the 5’ -terminus (ssDNA) was used as control (left panel). TBE-agarose gel (4%) analysis of the nuclease activities of TREX1 (5, 50, and 500 nM) in digesting ssDNA(m) and ssDNA (right panel). (**J**) The schematic design of double-stranded DNA containing 3mC at the 3′ -terminus and FAM-labelled at the 5 ′-terminus is depicted as dsDNA(m). Double-stranded DNA without 3mC at the 3′ -terminus and FAM-labelled at the 5 ′-terminus (dsDNA) was used as control (left panel). TBE-agarose gel (4%) analysis of the nuclease activities of TREX1 (5, 50, and 500 nM) in digesting dsDNA(m) and dsDNA. DNA ladder represents an ultra-low range size marker (NEB). All data represent mean ± S.E. (error bars) (*n*=5). *P<0.05 (Student’s t-test). 1mA, *N-*1-methyladenine; 3mC, *N-*3-methylcytosine; TERX1, three-prime repair exonuclease 1.

We observed that some MMS-treated DNA was degraded by TREX1, albeit poorly. To find the mechanistic explanation, we studied the effect of the 3meC present at different locations, namely, at the 3′-overhang region or internally within the duplex DNA. Having found that both 1mA and 3mC are inhibitory to TREX1 (Figure 3), we decided to use 3mC for this analysis. We designed and synthesized stem-loop duplex oligonucleotide to have a duplex region and also a single 5′ and 3′-termini. The 3′-end had a single-strand overhang to mimic the preferred conformation of the TREX1 substrate. One of the stem-loop oligonucleotides had two 3mC adducts flanking a regular cytosine in the middle within the stem region of the duplex (internal adduct) (Figure 4A). The other stem-loop oligonucleotides contained four tandem 3mC adducts in the 3′-overhang region (terminal adduct) (Figure 4E). DNA degradation was monitored by an SG-based ‘turn-off’ fluorescence assay. The presence of duplex conformation in either of the stem-loop structures was confirmed by CD spectroscopy (Figure 4B and F). As a negative control, TREX1 was incubated with stem-loop DNA having normal nucleotides. It was observed that the depletion of SG fluorescence by TREX1 was more in undamaged stem-loop DNA than in stem-loop DNA containing 3mC adduct at the 3′-overhang region (Figure 4C). The difference in SG fluorescence due to the presence of terminal 3mC was found to be significant (*P*<0.05) (Figure 4D). Using this assay, we further examined the effect of internally located 3mC adducts on TREX1 activity. It was observed that SG fluorescence of 3mC containing DNA changed almost similar to control stem-loop DNA without 3mC (Figure 4G), and the difference in SG fluorescence due to the presence of internal 3mC was not significant (Figure 4H). Notably, our results with MMS-damaged plasmid DNA (Figures 1 and 2) showed limited degradation of methylated DNA by TREX1. The data presented in Figure 4 suggest that the presence of methyl adducts in the duplex region (internal adduct) is unlikely to inhibit TREX1.

The activity of TREX1 and other DEDDh exonucleases may be influenced not only by the type of the base but also by DNA structure [33]. The presence of 3mC in the middle of a stem-loop DNA could alter the DNA structure, which in turn may affect TREX1 activity. It is possible that only two 3mC bases in the middle of the stem-loop were not enough to alter the structure. In MMS-treated plasmid DNA, multiple internally located 3mC or 1mA might alter the DNA structure and prevent the TREX1 activity.

Experiments with SG-bound stem-loop DNA showed the inhibitory effect of methyl adducts at the 3′-overhang region. However, SG binding could be influenced by DNA structure and the length of double-stranded regions, which could further affect the outcome. To obtain direct evidence to show that TREX1 preferentially targets normal DNA over DNA with methyl adducts, 5′-FAM-labelled single-stranded oligo-deoxynucleotide (ssDNA) containing multiple 3mC adducts at the 3′-end was synthesized. When this DNA was used as a TREX1 substrate, the 5′-FAM-labelled DNA was completely degraded at a higher concentration (500 nM) of TREX1 as visualized directly in agarose gels under UV light (Figure 4I). However, incomplete digestion was observed at lower concentrations (5 and 50 nM) of TREX1. Normal 5′-FAM-labelled control ssDNA was completely degraded by TREX1 (Figure 4I). Next, 5′-FAM-labelled stem-loop DNA was synthesized, which could be annealed to form duplex DNA with 3′-overhang with or without 3mC adducts. It was observed that stem-loop duplex DNA with 3mC adducts at the 3′-terminal resisted degradation at higher TREX1 concentrations (50 and 500 nM), but control stem-loop DNA without 3mC was completely degraded (Figure 4J). At lower TREX1 concentration (5 nM), control ssDNA was completely digested (Figure 4I), but control stem-loop duplex DNA was not (Figure 4J). These data suggest that TREX1 exhibits higher activity in digesting ssDNA and supports our earlier result with SG fluorescence and indicates that the presence of 3mC at the 3′-terminal was inhibitory to TREX1 activity.

## Discussion

Exposure of DNA to alkylating agents, such as MMS, results in a variety of alkyl adducts in the single-stranded and double-stranded DNA. Spontaneous removal of some of the damaged bases (e.g. 7 mG) generates DNA nicks. DNA nicks may be formed during BER. The DNA nicks provide an opportunity for the removal of a DNA strand by a 3′-5′ exonuclease. Such gapped DNA intermediate can be mended by repair synthesis and ligation [34]. Thus, 3′-5′ exonucleases not only generate a ‘clean’ 3′ hydroxyl group but also remove hundreds of nucleotides of damaged bases. Interestingly, the human genome encodes many well-characterized 3′-5′ exonucleases that are directly or indirectly involved in DNA damage response [35]. These include apurinic/apyrimidinic endonuclease 1 (APE1), TREX1, EXD2 (exonuclease domain-containing protein 2), tumour suppressor protein p53, MRE11, RAD1, RAD9, and WRN (Werner syndrome protein) [36]. The effect of adducts on APE1 activity was also studied previously [37,38]. However, which of these enzymes is capable of removing alkyl-nucleotide adducts from the 3′-overhangs was not known. We examined here whether the two cytotoxic methyl adducts, namely, 1mA and 3mC, are processed by TREX1. Three distinct methods were used to evaluate TREX1 activity on methylated DNA, namely, gel electrophoresis, SG fluorescence, and high-performance liquid chromatography (HPLC). While the gel electrophoresis and SG fluorescence could be used to quantitatively measure TREX1 activity, it could not answered whether one of the strands (methylated or unmethylated) of DNA in duplex was degraded or both the strands were degraded. Analysis of the product by HPLC provided powerful qualitative data on the release of methylated and unmethylated nucleotides. Our initial observation with the MMS-damaged DNA showed that MMS-treated methylated DNA was a poor substrate for TREX1 (Figures 1 and 2). Because MMS treatment produces a variety of methyl adducts, we determined the identity of the methyl adducts, i.e. 1mA and 3mC, which are inhibitory to TREX1 (Figure 3). We were also intrigued by the weak TREX1 activity that was observed against MMS-damaged DNA and wanted to investigate the mechanism. We found the presence of 3mC adduct in the present at the single-stranded 3′-termini to be particularly inhibitory for TREX1 activity (Figure 4). From the TREX1 structures in complex with various substrates reported [15,33,39], we know that the Leu24-Pro25-Ser26 cluster in TREX1 acts as a wedge and promotes terminal-unwinding of the scissile and the non-scissile strands. Another narrow pocket of the TREX1 active site binds the last nucleotide at the 3′-end by stacking interaction with Leu24 and Ile84. The presence of some DNA base adducts including 8-oxoG at the 3′-end could hinder the enzyme’s ability to stack bases efficiently; similarly, the abasic site would completely lack base-stacking interaction between the 3′-end of the scissile DNA strand and TREX1 [15]. From this structural insight, we propose that the presence of 1mA or 3mC-adducts at the scissile 3′-end might affect the capture of the 3′-end of the scissile strand, ultimately inhibiting the activity of TREX1. It remains to be explored whether TREX1 might participate in the removal of the alkyl-adduct containing DNA segment and generating gapped-DNA intermediate *in vivo*. Interestingly, TREX1 partners with PCNA and poly(ADP-ribose) polymerase-1 (PARP1) and is speculated to participate in the repair of ssDNA breaks [22,40]. However, having a limited ability to remove 1mA and 3mC, TREX1 might not contribute to the removal of unannealed DNA tails containing methyl adducts during the replication or recombination process. It is established that Fe(II)/2OG-dependent dioxygenase enzymes ALKBH2 and ALKBH3 remove various methyl adducts, including 1mA and 3mC, from double-stranded and ssDNA, respectively [41]. Contradictory to its name, which suggests a nuclear repair function, mutant TREX1 lacking TMD disrupts DNA damage repair by degrading 3′-overhangs, leading to the accumulation of deletions, cell death, and senescence [42]. Because TREX1 can also cause DNA damage, it is typically excluded from the nucleus. Based on the results presented here, it seems that TREX1 exonuclease may be capable of resecting DNA with 1mA and 3mC adducts from the 3′-end, provided that the 3′-terminal DNA is undamaged.

## Materials and methods

### Cloning, mutagenesis, expression, and purification of TREX1 and TREX1 mutant

N-terminal 242 amino acid residues of TREX1 (lacking the C-terminal ER-anchoring domain) were PCR amplified from the cDNA and cloned in pET28a vector to generate in-frame hexa-histidine tag at the N-terminus (**online supplementary material 1, online supplementary figure 1**). Catalytically inactive mutant TREX1 H195A was generated by site-directed mutagenesis as suggested by Brucet et al. [31]. Recombinant His-tag TREX1 and TREX1 H195A mutant were purified using Ni-NTA chromatography and further purified by SEC (FPLC) (Supplementary information).

### MMS treatment of plasmid DNA

Methylated plasmid DNA was generated as described previously [43]. In brief, 10 μg of plasmid DNA (pBlueScript) was linearized with SacI (NEB) restriction enzyme and treated with 5% (v/v) (0.59 M) MMS (Sigma) in a final volume of 500 μl for 16 h at room temperature. The methylated DNA was not purified directly by using ethanol precipitation, as it resulted in poor yield. Therefore, excess MMS was removed by dialysis against TE buffer (10 mM Tris, pH 8.0, 1 mM EDTA) using Spectra/Por dialysis membrane (MWCO: 1 kDa) for 12 h. Then, the damaged DNA was precipitated by adding 0.3M sodium acetate pH 5.5 and 2 volumes of ice-cold ethanol. The precipitated methylated DNA was washed with 70% ethanol and finally dissolved in molecular-grade water.

### Detection of TREX1 activity by agarose gel electrophoresis

TREX1 assay was performed as reported earlier with some modifications [30]. The TREX1 substrate was prepared by linearizing the pBlueScript plasmid with the SacI (NEB) restriction enzyme. The substrate DNA (5 ng/µl) was incubated with the recombinant wildtype or mutant TREX1 H195A (1 µM) in the presence of reaction buffer (2 mM DTT, 50 mM pH 7.5 and 5 mM MgCl_2_) at 37°C for 1 h. Reactions were stopped by adding 20 mM EDTA at different time points and analysed by agarose gel electrophoresis and ethidium bromide staining. For chemically synthesized 30-mer duplex oligonucleotide, unmethylated dT-oligonucleotide was annealed with (dA-d1mA)_15_ oligonucleotide, and unmethylated dG-oligonucleotide was annealed with (dC-d3mC)_15_ oligonucleotide. The annealed DNA was incubated with TREX1 (1 µM) for 30 min at 37°C. The reaction mixture was run in 4% Agarose (Lonza-50004) gel prepared in 1X Tris-acetate EDTA buffer 110V for 20 min. The gel image was taken using Syngene UV transilluminator Gel-doc, and the bands were further quantified using ImageJ software.

### Fluorescence-based TREX1 assay

The SG fluorescence-based TREX1 assay was performed as reported earlier with some modifications [44]. Recombinant wildtype and mutant TREX1 H195A and the DNA substrate were prepared in the reaction buffer as mentioned previously. The degradation of DNA was monitored by adding 1X SG solution. Fluorescence was measured using a BioTek Synergy H1 microplate reader (λ_ex_=305 nm, λ_em_=363 nm, gain 100, kinetic interval 5 min).

### Fluorescence-based TREX1 assay using hairpin DNA substrate containing 3mC

The DNA oligonucleotides containing 3mC at specific locations were chemically synthesized (GeneLink Inc., USA). For stem-loop/hairpin DNA, the sequence of the oligonucleotide was as follows: 5′-AAATCGTTGC CTCCTTCGAG CGCGCAAAGA ATTAAAGAGC TCTTAGTCAG AAGGATACTA AGAGCTCTTT AATTCTTTG[3mC] G[3mC]GCTCGAAG GAGGCAACGA TTTC3′. For terminal damage, 5′-AGGGTTAGGG TTAGGGTTAG GGCAGAAGGA TAACCCTAAC CCTAACCCTA ACCCT[3mC][3mC][3mC][3mC]-3′. For 5′-FAM labelled oligo DNA, the sequence was 5′-FAM-AAAAAAAAAAAAAAAAAAAAAAAA CAGAAGGATAA TTTTTTTTTTTTTTTTTTTTTTTT-[3mC][3mC][3mC][3mC]-3′ and for ssDNA oligo was 5′FAM-TTTTTTTTTTTTTTTTTTTT-[3mC][3mC][3mC][3mC]-3′. Annealing of the stem-loop DNA (10 µg) was carried out in annealing buffer (10 mM Tris-HCl, pH 8.0) and 50 mM NaCl. The DNA was heated to 95°C for 10 min and cooled at a rate of 0.1 °C/s to reach 4°C. This stem-loop DNA was incubated with TREX1 (5–500 nM) in TREX1 reaction buffer at 37°C for 30 min. Following this, 1× SG was used to monitor the DNA degradation. Following the electrophoresis of FAM-labelled samples, images were captured in the GelDoc-Go (Bio-Rad) system. For SG-labelled samples, readings were taken at 30-min intervals wherein the excitation was set at 480 nm and the emission range was 510–650 nm.

### HPLC analysis of TREX1 reaction

Chemically synthesized 30-mer oligonucleotides containing dA, dG, dC, or dT were dissolved in water (100 µM). Oligonucleotides containing dA and dC were methylated by treating with MMS (5%, v/v) (0.59 M) (Sigma, 129925) and purified as described previously [45]. For the double-stranded DNA substrate, unmethylated dT-oligonucleotide was annealed with 1mA containing dA-oligonucleotide, and unmethylated dG-oligonucleotide was annealed with 1mC containing dC-oligonucleotide. For determining TREX1 activity, TREX1 (0.5 μM) was mixed with single-stranded or double-stranded oligonucleotide or (1 µg) in TREX1 reaction buffer incubated at 37°C for 20 min, and the reaction was stopped by adding 25 mM EDTA. The dNMPs were analysed in an HPLC system (Shimadzu) equipped with a reverse-phase Shim-pack GIST C18 5 µm separation column (250 × 4.6 mm). For 1me-dAMP, dAMP, dTMP and 3me-dCMP, dCMP, dGMP, the mobile phase is the same for both cases, that is, mobile phase A (50 mM ammonium acetate) and mobile phase B (50 mM ammonium acetate, 50% of acetonitrile and 0.1% trifluoroacetic acid [TFA]). The peaks were identified based on the elution time of the standards (Sigma) (Supplementary Figure S2) and further analysed using LabSolutions software.

## Supplementary material

online supplementary figure 1.

online supplementary figure 2.

online supplementary material 1.

## Data Availability

All the data supporting this article have been included as part of the manuscript. Any additional data will be made available upon request to the corresponding author.

## Abbreviations

BER: base excision repair
HPLC: high-performance liquid chromatography
MMS: methyl methanesulfonate
TERX1: three-prime repair exonuclease 1
TMD: transmembrane domain
cGAS-STING: cyclic GMP-AMP synthase-stimulator of interferon genes
1mA: *N*-1-methyladenine
3mC: *N*-3-methylcytosine
