# Supplementary figures and images for: Effect of methyl DNA adducts on 3’-5’ exonuclease activity of human TREX1
Source: Biochem J. 2025 Mar 5;482(5):BCJ20240600. doi: 10.1042/BCJ20240600 (PMC12133304; doi:10.1042/BCJ20240600)

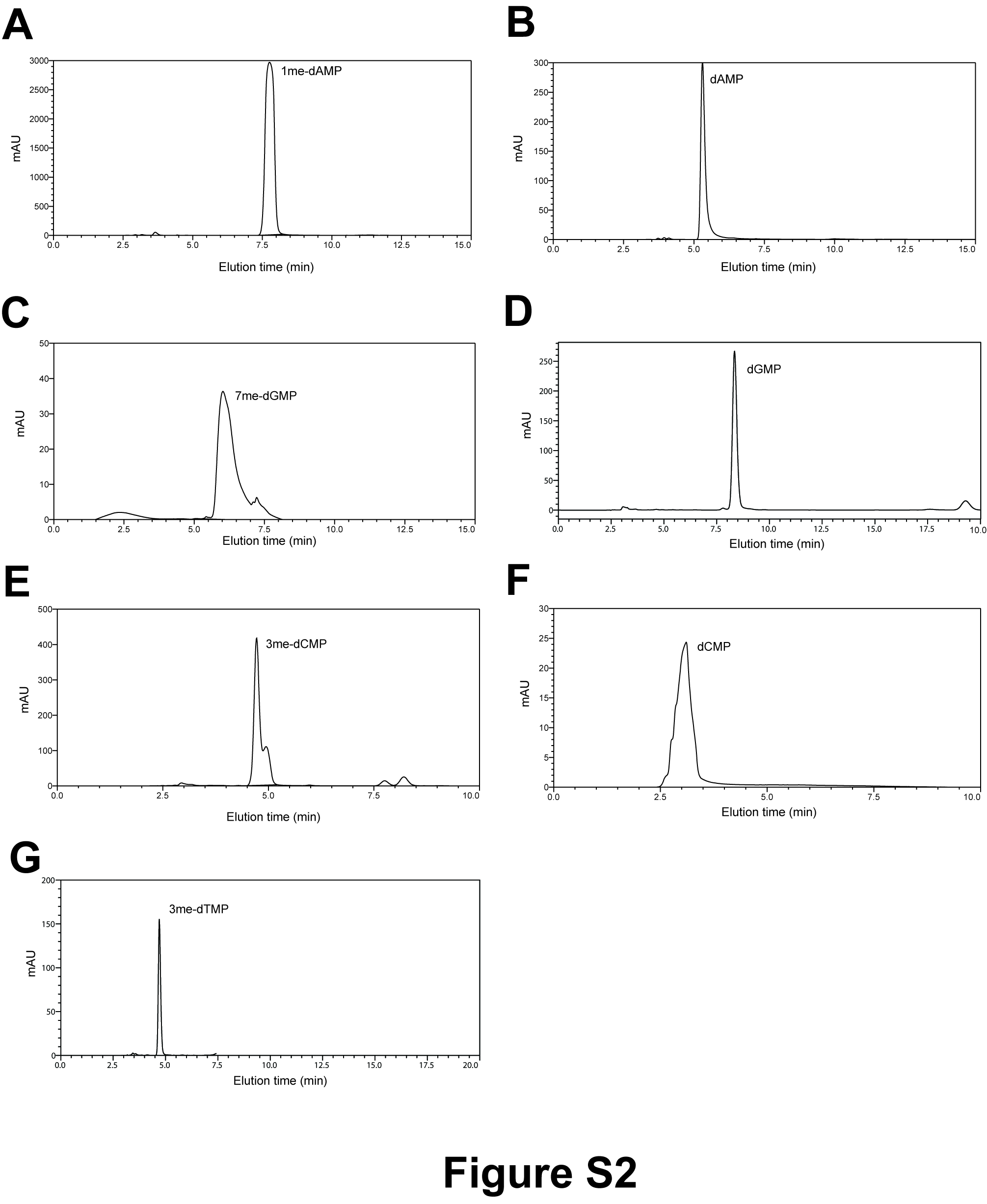

Supplement: online supplementary figure 1. [file bcj-482-5-BCJ20240600-s003.tif]

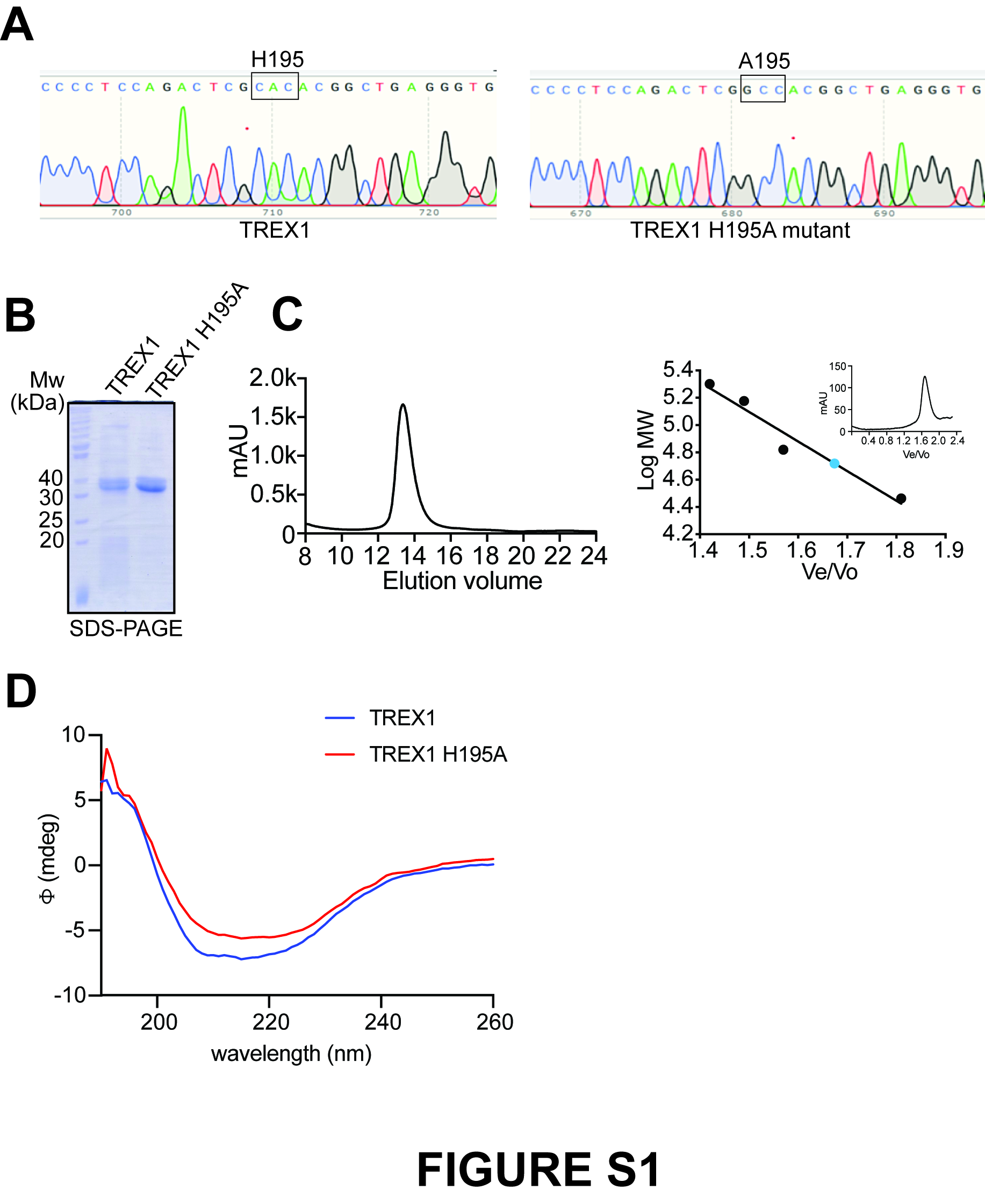

Supplement: online supplementary figure 2. [file bcj-482-5-BCJ20240600-s001.tif]
