# Supplementary material for: Effect of methyl DNA adducts on 3’-5’ exonuclease activity of human TREX1
Source: Biochem J. 2025 Mar 5;482(5):BCJ20240600. doi: 10.1042/BCJ20240600 (PMC12133304; doi:10.1042/BCJ20240600)
Supplement: online supplementary material 1. [file bcj-482-5-BCJ20240600-s002.docx]

**Effect of methyl DNA adducts on 3’-5’ exonuclease activity of human TREX1**

Nikhil Tuti^1^, Unnikrishnan P Shaji^1^, Susmita Das^1^, Roy Anindya^1,^*

**Supplementary Methods**

**Cloning, expression, purification of TREX1, and TREX1 H195A mutant:** TREX1 coding sequence lacking the C-terminal residues (N-terminal 242 amino acid residues) of TREX1 was PCR amplified using a specific forward primer (5’ ATA AGC TAG CAT GCA GAC CCT CAT CTT TTT CGA CAT GGA G 3’) and reverse primer (5’ TAT TCT CGA GTT ATG CAG TGG TTG TGA CAG CAG ATG GTC T 3’). Human PBMC cDNA was used as the template for the cDNA amplification. PCR amplified product was cloned in pET28a vector to generate His-tag TREX1. For this, the amplified PCR product and pET28a plasmid were digested with NheI and Xhol and ligated with T4 DNA ligase with T4 DNA ligase (NEB M0202S). Then the ligated product was transformed into DH5-alpha cells and the clones were confirmed by sequencing (**Figure S1 A**).

Mutant TREX1 H195A was generated by site-directed mutagenesis. For this, a mega-primer was generated using PCR. The primers used for generating mega-primer were: forward primer- 5’ TCC CCT CCA GAC TCG GCC ACG GCT GAG GGT GAT 3’; reverse primer 5’ TTA TGC AGT GGT TGT GAC AGC AGA TGG TCT TGG 3’. Then this mega-primer containing mutation was used for mutagenesis PCR using TREX1 plasmid as template. Then the PCR product was treated with DpnI and transformed into the DH5-alpha cells. The mutant clones were then confirmed by sequencing (**Figure S1 A**).

Recombinant His-tag TREX1 and TREX1 H195A mutant were purified using Ni-NTA chromatography following manufacturer instructions. The protein was finally dialysed in a buffer containing 50 mM Tris-HCl, pH 8.0 and 100 mM KCl. Further, the purity of His-tag TREX1 and TREX1 H195A was confirmed by 10% SDS-PAGE (**Figure S1 B**). The recombinant TREX1 was further purified by size exclusion chromatography (SEC). SEC was performed in FPLC instrument (Aktapure GE Healthcare) using Superose 12 10/300 GL column. The column was equilibrated with buffer containing 50 mM Tris pH 8.0 and 100 mM KCl and 500 µg of TREX1 was injected into the column at a flow rate of 0.2 ml/min. Then the elution time was recorded for the proteins. The total run time was 24 minutes (**Figure S1 C**). Finally, the folding of recombinant TREX1 was confirmed by circular dichroism (CD) analysis using Jasco J-1500. 1 mm quartz cuvttee (Starna scientific). For this, TREX1 was first diluted at 0.1 mg/ml and 200 µl was taken to the cuvette. The spectra were recorded as an average of three spectral scans with step 1 nm, 0.5 sec/point and bandwidth 1 nm. The scanning speed was 50 nm/sec, and the data pitch was set as 0.5 nm. The CD spectra were analysed using spectra manager software. The obtained CD spectre (ellipticity) was plotted against wavelength (**Figure S1 D**).

**Supplementary Figure Legends**

**Figure S1.** Cloning, mutagenesis and purification and characterization of recombinant TREX1 **(A)** Sequence analysis of TREX1 and TREX1 H195A mutant **(B)** SDS-PAGE analysis of purified TREX1 and TREX1 H195A. It should be noted that the protein loading is not equal for all proteins and differentially stained SDS-PAGE gels are depicted. **(C)** Analysis of TREX1 by size exclusion chromatography (left panel). The chromatogram was used to calculate V_e_/V_0_​, which then used estimate the molecular weight (MW) of the protein using the standard plot shown in the right panel. The blue circle represents truncated His-TREX1 and its MW was determined to be approximately 53.23 kDa, closely matching the double of theoretical MW of 55 kDa, suggesting that TREX1 exists as a dimer. **(D)** Circular dichroism (CD) analysis of TREX1 and TREX1 H195A.

**Figure S2.** Retention of a series of standard nucleotide compounds on a reverse-phase high-performance liquid chromatography (HPLC) column (Shim-pack GIST C18 5 µm column (250 x 4.6 mm) flow rate 1 ml/min).­­­ (A) 1me-dAMP (7.76 min) (B) dAMP (5.3 min) (C) 7me-dGMP (6.02 min) (D) dGMP (8.34 min) (E) 3me-dCMP (4.72 min) (F) dCMP (3.10 min) (G) dTMP (4.7 min).
